# Venous Access: National Guideline and Registry Development (VANGUARD): Advancing Patient-Centered Venous Access Care Through the Development of a National Coordinated Registry Network

**DOI:** 10.2196/43658

**Published:** 2023-11-24

**Authors:** Andrea Iorga, Marti J Velezis, Danica Marinac-Dabic, Robert F Lario, Stanley M Huff, Beth Gore, Leonard A Mermel, L Charles Bailey, Julia Skapik, Debi Willis, Robert E Lee, Frank P Hurst, Laura E Gressler, Terrie L Reed, Richard Towbin, Kevin M Baskin

**Affiliations:** 1 Center for Devices and Radiological Health US Food and Drug Administration Silver Spring, MD United States; 2 Computer Science and Electrical Engineering University of Maryland, Baltimore County Baltimore, MD United States; 3 Biomedical Informatics Research University of Utah Salt Lake City, UT United States; 4 Biomedical Informatics University of Utah School of Medicine Salt Lake City, UT United States; 5 The Oley Foundation Albany Medical Center Delmar, NY United States; 6 Division of Infectious Diseases Department of Medicine Warren Alpert Medical School at Brown University Providence, RI United States; 7 Biomedical and Health Informatics Children’s Hospital of Philadelphia Philadelphia, PA United States; 8 Pediatrics Perelman School of Medicine University of Pennsylvania Philadelphia, PA United States; 9 Internal Medicine Inova Medical Group Alexandria, VA United States; 10 National Association of Community Health Centers Bethesda, MD United States; 11 PatientLink Enterprises Oklahoma City, OK United States; 12 Pharmaceutical Evaluation and Policy University of Arkansas for Medical Sciences Little Rock, AR United States; 13 Symmetric Health Solutions Pittsburgh, PA United States; 14 Emeritus, Department of Radiology Phoenix Children’s Hospital Phoenix, AZ United States; 15 VANGUARD Coordinated Registry Network, LLC Phoenix, AZ United States; 16 Division of Interventional Radiology Department of Radiology Conemaugh Memorial Medical Center Johnstown, PA United States

**Keywords:** central venous access devices, registry, patient-reported outcomes, catheter, CRBSI, CLABSI, development, patient, therapy, life-threatening, clinical, reliable, policy, system, medical device

## Abstract

There are over 8 million central venous access devices inserted each year, many in patients with chronic conditions who rely on central access for life-preserving therapies. Central venous access device–related complications can be life-threatening and add tens of billions of dollars to health care costs, while their incidence is most likely grossly mis- or underreported by medical institutions. In this communication, we review the challenges that impair retention, exchange, and analysis of data necessary for a meaningful understanding of critical events and outcomes in this clinical domain. The difficulty is not only with data extraction and harmonization from electronic health records, national surveillance systems, or other health information repositories where data might be stored. The problem is that reliable and appropriate data are not recorded, or falsely recorded, at least in part because policy, payment, penalties, proprietary concerns, and workflow burdens discourage completeness and accuracy. We provide a roadmap for the development of health care information systems and infrastructure that address these challenges, framed within the context of research studies that build a framework of standardized terminology, decision support, data capture, and information exchange necessary for the task. This roadmap is embedded in a broader Coordinated Registry Network Learning Community, and facilitated by the Medical Device Epidemiology Network, a Public-Private Partnership sponsored by the US Food and Drug Administration, with the scope of advancing methods, national and international infrastructure, and partnerships needed for the evaluation of medical devices throughout their total life cycle.

## Introduction

### Overview

Central venous access care involves a complex nexus of patients, providers, health institutions, payers, regulators, and other critical stakeholders (Textbox 1). An estimated 8 million central venous catheters inserted each year in the United States generate tens of billions of dollars in added costs from related complications [1-3]. A lack of meaningful and accurate outcomes data and limited accountability for related care remain critical challenges to successful improvements in care and reduction of harm. Absence of a medical specialty routinely responsible for patients after the implantation of a central venous access device (CVAD) further restricts the ability to address these challenges. Even national surveillance stops at the hospital door. The purpose of this communication is to describe a roadmap for developing the Venous Access: National Guideline and Registry Development (VANGUARD) Coordinated Registry Network (CRN) to meet these challenges and thus improve the quality of care provided to patients requiring CVADs.

**Venous Access: National Guideline and Registry Development (VANGUARD) Coordinated Registry Network (CRN) Stakeholder Community. The VANGUARD CRN community includes representatives from the following stakeholder groups.**

**Parents and families**

- Oley Foundation
- Short Gut Syndrome Families’ Support Group
- VANGUARD patient advocacy committee

**Nursing specialties**

- Ward nursing
- Infusion nursing
- Apheresis nursing
- Home health nursing
- Emergency department nursing
- Peripherally inserted central catheter (PICC) nursing
- Intestinal care nursing
- Oncology nursing
- Advanced practice nursing
- Nurse anesthetists

**Physician specialties**

- Interventional radiology
- General surgery
- Interventional nephrology
- Interventional cardiology
- Vascular surgery
- Critical care medicine
- Anesthesiology
- Emergency medicine
- Infectious disease medicine
- Hematology or oncology
- Gastroenterology

**Allied health professions**

- Respiratory care
- Pharmacy or pharmacology
- Coding and billing
- Patient liaison
- Discharge planning

**Medical specialty societies and core groups**

- Society for Interventional Radiology
- Medical Device Epidemiology Network
- National System for Evaluation of Health Care Technology
- Cardiovascular and Interventional Radiology Society of Europe
- Society of Health Care Epidemiology of America
- Health Care Infection Control Practices Advisory Committee
- Institute for Health Care Improvement
- American Pediatric Surgical Association
- Association for Professionals in Infection Control and Epidemiology
- Infusion Nurses Society
- American College of Surgeons
- American Association of Critical Care Nurses
- American Society of Anesthesiologists
- American Society of Diagnostic and Interventional Nephrology
- American Society of Parenteral and Enteral Nutrition
- American Society of Transplant Surgeons
- Alliance for Vascular Access Teaching and Research
- European Society for Clinical Nutrition and Metabolism
- Home Care Association of America
- Infectious Disease Society of America
- International Pediatric Transplant Association
- International Society of Thrombosis and Hemostasis
- Interventional Radiology Society of Australasia
- National Association of Community Health Centers
- North American Society for Pediatric Gastroenterology, Hepatology, and Nutrition
- Oncology Nursing Society
- Pediatric Infectious Diseases Society
- Vascular Access Society of the Americas
- Visiting Nurse Association

**Federal agencies and offices**

- US Food and Drug Administration

**US Department of Health and Human Services**

- Centers for Disease Control and Prevention
- Agency for Health Care Research and Quality
- Office of the National Coordinator for Health Information Technology
- Centers for Medicare and Medicaid Services
- National Institutes of Health
- National Library of Medicine
- National Cancer Institute (Data Standards Registry & Repository)

**Device manufacturers**

- Abbott
- Angiodynamics
- Argon
- Arrow
- Avenu
- Bard or Becton-Dickinson
- Cook
- MedComp
- Merit Medical

**Health care information technology**

- Logica
- First Databank
- Perspecta
- Epic
- Cerner
- Allscripts
- Intelligent Medical Objects
- Quintiles
- IQVIA
- Symmetric Health Solutions
- Medstreaming

**Payers**

- Blue Cross-Blue Shield
- United Health Group
- Anthem
- Cigna
- Aetna

**Private agencies**

- PEW Charitable Trusts
- Brookings Institute
- Health Level 7
- National Patient-Centered Clinical Research Network (PCORnet) or Pediatric Research Network (PEDSnet)
- The Joint Commission
- Physician Consortium for Performance Improvement
- Centers for Medicare and Medicaid Services (CMS) Alliance to Modernize Health care

### Background

CVAD complications carry significant risks and may delay treatment, damage vessels, limit other vascular access options, cause pain, decrease quality of life, and increase morbidity and mortality [4-7]. The most important of these harms, catheter-related bloodstream infection (CRBSI), is so poorly studied that the upcoming Infectious Disease Society of America guidelines were not able to identify many well-constructed, properly controlled randomized CRBSI studies despite comprehensive reviews of available literature (personal communication: KMB and LAM).

Per clinical practice guidelines, accurate and meaningful diagnosis of CRBSI requires (1) a health care provider responsible for and capable of recognizing the signs and symptoms of an infection, (2) microbiologic evidence of a local or bloodstream infection, and (3) exclusion of a noncatheter source of the infection. In essence, all central venous catheter patients manifesting signs and symptoms of infection should have blood cultures obtained, or, if present, a swab culture of any purulent exit site drainage, and an evaluation should be performed to exclude an alternate source of infection to explain a patient’s symptomatology. If the catheter must be removed (eg, due to hemodynamic instability), the catheter tip should be sent for culture. If such steps were followed, an unequivocal diagnosis of CRBSI could be achieved or excluded in most cases [8]. Even presumed experts often do not follow these steps [9].

Medical providers may remove the catheter presumptively and may or (according to the VANGUARD Affected Persons panel) may not offer antibiotic therapy. They may believe they have treated the infection and removed its source, thus fulfilling their clinical responsibilities. Further, they can avoid the risk of reporting a health care–associated infection and therefore avoid potential economic penalties and public relations fallout. They have streamlined workflow, minimized documentation overhead, and avoided the need for follow-up care. However, reportable events may not have been captured, patients may have been mistreated, and may have been left without a vital route of access for life-preserving therapy. It is challenging for providers and institutions to avoid such potentially harmful actions without leaving the affected patients to bear the consequences [1,10]. In a recent unpublished survey of 470 patients who have had at least one central line placed in their lifetime for the treatment of chronic diseases, nearly 80% have had one or more catheter complications, and almost 60% have had at least one catheter-related infection [11]. Over 40% of these affected patients did not believe their health care providers knew how to properly take care of their catheter-related concerns.

Central line–associated bloodstream infection (CLABSI), the adjunct epidemiologic metric, is one of the key health care–associated infections recognized as a national public health priority, associated with high rates of morbidity and mortality [12-15]. As a major health care concern, CLABSI is complex and multifaceted, and has thus far defied efforts to derive a successful standard solution or even well-defined guidelines supported by objective and valid data [15]. This is likely because CLABSI is rife with such methodologic, economic, and political bias as to render it in practice a very low-quality variable poorly suited to the identification of trends or the prevention of disease [1]. This problem is exacerbated by the fact that the majority of catheter-related complications may occur outside of the venues where CLABSI are monitored, including emergency and urgent care facilities, long-term care facilities, and patients’ homes [16]. Furthermore, existing health care information systems do not facilitate access to cross-institutional, cross-specialty, or cross-proprietary systems and software required to collect critical venous health history data [17]. There is a lack of controlled, multidisciplinary, and multicenter evidence, which renders decisions based on current guidelines of uncertain value for patient-centered outcomes [18].

The Centers for Disease Control and Prevention estimates more than 270,000 CLABSI cases occur in the US annually [12,19] in spite of the 2011 Centers for Disease Control and Prevention-issued multidisciplinary Guidelines for the Prevention of Intravascular Catheter–Related Infections (CRBSI) [20]. This document included recommendations on staffing, catheter site selection, skin preparation and dressing regimens, hand hygiene, and aseptic technique. Similar suggestions for evidence-based practices soon followed, resulting in an estimated 46% reduction in CRBSI cases [12,21-23], although very recently available data suggests that the number of cases may again be on the rise [12]. These conflicting reports may be expected given that the methodology for measurement of CLABSI remains highly flawed [1,24,25]. For example, measurement of CLABSI and diagnosis of CRBSI both rely on the appropriate acquisition and interpretation of cultures. With the imposition of CLABSI-related penalties, the frequency of blood cultures obtained in central catheter patients fell precipitously [26]. Considering the number of patients who have complications outside of the hospital, combined with the unreported and misreported cases of CRBSI, CLABSI estimates seem to grossly misrepresent the magnitude of patient harm. Clearly, more comprehensive data of much higher quality is not just a theoretical public health priority, it is an urgent necessity. Successful solutions must account for the simultaneous fulfillment of the value propositions and conflicting priorities perceived by the many critical stakeholders in this domain (Textbox 1). This classic type of problem frequently encountered in the health care information technology ecosystem requires novel strategic solutions and “demands social processes that constantly engage stakeholders, explore related issues, reevaluate the problem’s definition, and reconsider the assumptions of stakeholders” [27].

### CRNs and the Medical Device Epidemiology Network

Medical Device Epidemiology Network (MDEpiNet), a Public-Private Partnership sponsored by the US Food and Drug Administration, has the scope of advancing methods, national and international infrastructure, and partnerships needed for the evaluation of medical devices throughout their life cycle. Under a cooperative agreement with the MDEpiNet Coordinating Center, the US Food and Drug Administration Center for Devices and Radiological Health has undertaken a collaborative effort to establish medical device CRNs with the objective of creating an interoperable infrastructure for gaining real-world evidence in 12 clinical areas. This effort was supported by the US Department of Health and Human Services Office of the Assistant Secretary for Planning and Evaluation Patient-Centered Outcomes Research Trust Fund Initiative. CRNs include comprehensive structured data elements and organized data systems that improve patient health care and add quality and efficiency across study designs within designated clinical domains [28,29]. CRNs are working to overcome the limitations of conventional registries which are bound to only one data source by utilizing a uniquely developed lexis to continuously extract current and relevant data elements and outcomes from diverse electronic health records (EHRs) and other data sources that may be external to contributing registries but still intrinsic to understanding the domain. However, truly interoperable solutions remain elusive [30-32].

### VANGUARD CRN

#### Approach

In 2014, the Society of Interventional Radiology sponsored a national multidisciplinary Research Consensus Panel (RCP) that prioritized issues and infrastructure needs for chronic CVAD patients. The RCP gave the highest priority to the development of a CVAD registry that unifies high-quality patient and device data across the device life span, across the patients’ disease cycle, and across relevant venues of care and complications and introduced the VANGUARD initiative as a cross-stakeholder medium to achieve these goals. Two subsequent multistakeholder symposia helped crystallize the VANGUARD mission and enumerate the tasks critical to its development [1].

During the RCP and following, it was recognized that there are some instances of CVAD registries either existing or in development on a hospital-wide basis, such as the Central Venous Access Device Registry of Royal Brisbane and Women’s Hospital in Queensland, Australia [33] and extensive data-gathering efforts on a state-wide [34] or medical specialty [35] basis. While important, such efforts have not met the criteria for a CRN, as described above, and have not achieved the level of data quality or generalizability required to meaningfully target policy, decision support, and quality interventions.

At the 2016 VANGUARD Stakeholder Symposium, it was agreed that initial efforts should center on compelling clinical, economic, and quality-of-life concerns in well-defined populations at risk that can be feasibly addressed within the time and resources available. Since the patients at highest risk of complications and those who consume the greatest proportion of health resources are those who require long-term access to the treatment of chronic diseases, VANGUARD proposed to deliver a secure and interoperable exchange of high-value health information and meaningful outcomes, and to leverage multidisciplinary, multi-institutional evidence for the purpose of improving patient-centered outcomes while reducing costs and complication rates related to chronic central venous access. From its inception, VANGUARD has been a multistakeholder-driven effort [1]. Following the publication of a landmark report by the National Medical Device Registry Task Force in 2015 that recommended the establishment of CRNs, VANGUARD joined other existing and aspiring registries to form a strategic foundation of CRNs in their respective clinical areas [36,37].

In parallel with the recruitment of numerous volunteers who helped clarify and coordinate aims and objectives across the stakeholder community, VANGUARD was adopted as one of the early enterprises within the MDEpiNet community with a pivotal role in the chronic venous access space [36,38]. Through extensive stakeholder partnerships, the initiative serves as part of national efforts to gather, synthesize, and evaluate information related to chronic CVADs, to improve medical device safety and effectiveness, and to sponsor terminology development and research in the central venous access domain.

#### The VANGUARD CRN Research Roadmap

In many health care domains, it is possible to access large volumes of low-quality data currently held in EHRs and other sources. However, low-quality data produces low-quality answers and may have zero or negative impact on patient-centered outcomes [39]. CLABSI, for example, is 1 such low-quality metric that is in widespread use but has yet to facilitate a “cure” for CRBSIs. As is also true in many health care domains, the high-quality data needed to demonstrate and ameliorate actual harm to patients is not currently collected and cannot be derived from existing data. Even the standardized terminology necessary to identify critical events and permit the exchange and extraction of meaningful data does not yet exist. VANGUARD shares these challenges of interoperability and data quality with all other existing and emerging CRNs. The VANGUARD CRN Research Roadmap has been designed to address these ecosystem-wide issues in a stepwise fashion, breaking down these challenges into achievable modules that build toward the desired end points while also leveraging resources developed by other CRNs.

**Figure 1:**
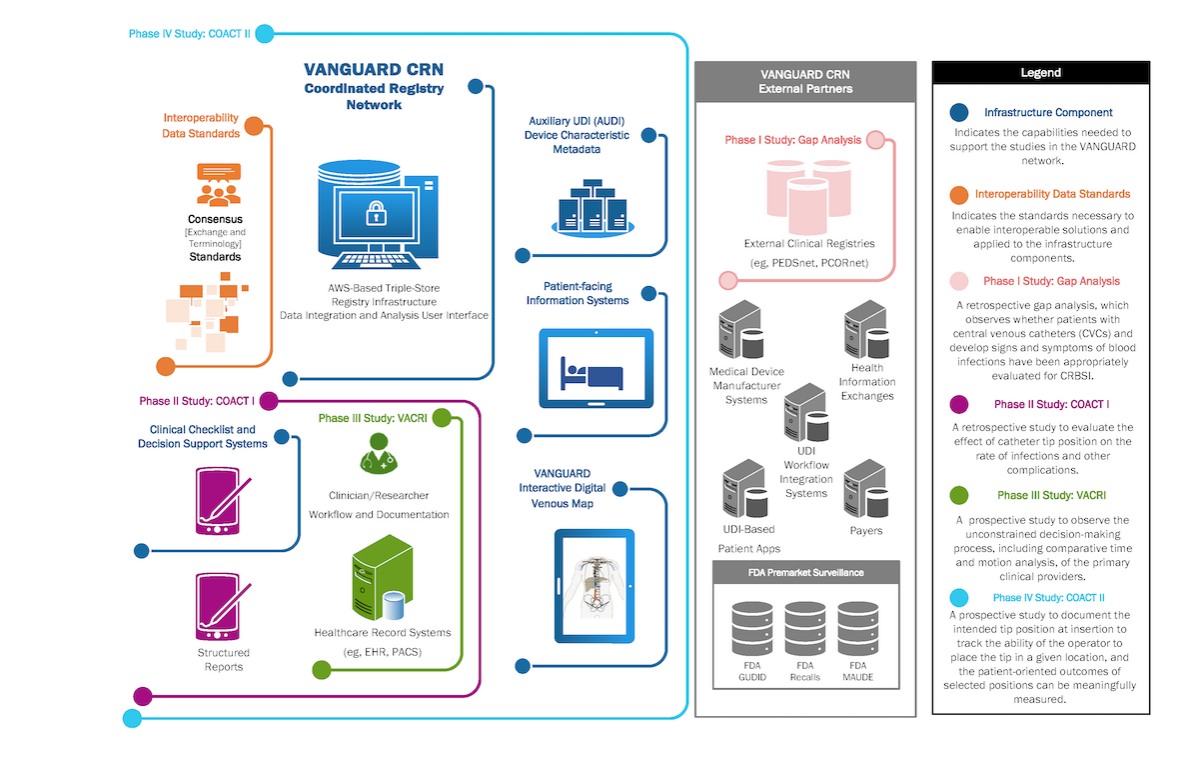
VANGUARD CRN Registry Roadmap. AUDI: auxiliary unique device identification; COACT I: clinical outcome analysis of catheter tip-position; COACT II: clinical outcome analysis of catheter tip-position II; CRBSI: catheter-related bloodstream infection; CRN: coordinated registry network; EHR: electronic health record; FDA: US Food and Drug Administration; PACS: picture archiving and communication system; PCORnet: National Patient-Centered Clinical Research Network; PEDSnet: Pediatric Research Network; UDI: unique device identifier; VACRI: venous access catheter-related infection; VANGUARD: venous access: national guideline and registry development.

The VANGUARD CRN has outlined several research studies designed to inform its further development, including (1) retrospective studies requiring databases of medical records and manual chart reviews, (2) prospective studies which incorporate structured reports and integrated decision support systems based on the results of the retrospective studies, and (3) complementary infrastructure development, which will support one or more of the prospective or patient engagement studies while providing decision-support tools that improve both interoperability and data quality. A cross-cutting objective for the VANGUARD activities is to identify the use of existing or emerging interoperability data standards and address any current gaps by contributing to their improvement. The end goal is to create and sustain a workflow-friendly set of transparent tools that facilitate accurate documentation and improved patient-centered outcomes without increasing the burden on clinicians (Figure 1). Since it is expected that these tools will have ubiquitous applications across the health care enterprise beyond chronic central venous access, it is the express intent of this initiative that the development, testing, and implementation of these tools occur in the most transparent and collaborative fashion possible, such that the work and the benefits will be shared across both CVAD stakeholders and the CRN Learning Community [38,40].

#### Retrospective Studies

The VANGUARD CRN hypothesizes that reports of downward trends in CLABSI may be misleading due to the compelling issues with data quality outlined above. The impression of many leading experts in the field is that CRBSI rates remain high, especially in populations of patients requiring chronic venous access and whose infections may manifest outside of the acute care setting [1]. It is important to establish how often chronic access patients presenting with signs and symptoms of infection are inadequately evaluated or misdiagnosed. The first phase of research on the VANGUARD agenda is a retrospective gap analysis, which will investigate whether patients with CVADs and suspected bloodstream infections have been appropriately evaluated for CRBSI. Two large databases [41,42] including over 77 million patient records will be interrogated for the co-occurrence of CVADs together with signs and symptoms of bacterial or fungal infection, such as fever, rigors, hypotension, lactic acidosis, nausea or vomiting, purulent discharge from the insertion or exit site, leukocytosis, and catheter removal associated with presumption of infection. Infectious disease experts assert that in practically all such cases, blood cultures should be obtained to validate the diagnosis [9,43]. A significant gap between the number of CVAD patient records exhibiting “prima facie” evidence of infection (or lacking sufficient data to assess for infection) and the number of records with concurrently obtained blood cultures would be indicative of the potentially harmful impact of low-quality data and nonstandard diagnostic practices on accurate diagnosis, documentation, and treatment of these high-risk patients. In this context, a missed or misreported bloodstream infection may be more deadly than one that is accurately diagnosed and appropriately treated.

The second retrospective study, Clinical Outcome Analysis of Catheter Tip-position-I (COACT I), will evaluate the effect of catheter tip position on the rate of infections and other complications. This multi-institutional study will include up to 1500 patients with indwelling ports, and will reference pre- and intraprocedural data, as well as postprocedural longitudinal outcomes data and contemporaneous x-ray images. COACT I will test the hypothesis that the safest place to position a catheter tip can be unequivocally determined using high quality retrospective data. Secondary objectives of this study include the effect of catheter tip position on clinical outcomes (ie, incidence of thrombotic outcomes, infectious outcomes, mechanical complications, and catheter-related deaths), effect of side or site of insertion, and effect of previous history of poor or difficult access. The findings of this study will greatly impact a subsequent phase, COACT II, which will put into practice the insights gained here.

#### Prospective Studies

For any meaningful comparison of clinical practices to be performed, there must be a reliable standard of truth available to measure performance. In the context of the studies described above, this requires a reliable anatomic standard for the description of catheter tip position and a reliable outcome measure for complications such as CRBSI. Currently, neither exists in general practice. The former challenge has been addressed by prior VANGUARD-led research that developed and established the accuracy of methodology that uses structures visible on a plain chest radiograph to unequivocally describe the central access tip position [44]. Related research is ongoing within the VANGUARD suite designed to provide an accurate and easily applied documentation bridge between such descriptions and terminology in common use.

Meaningful patient-centered outcome measures will require clinicians to comply with diagnostic clinical pathways that provide the data points necessary for accurate measurement. There are 2 additional components that will be necessary for successful implementation of the best practices revealed through this process. First, the professional autonomy of clinical decision makers must be respected. Second, the implemented process must not add to the clinicians’ workflow and documentation burden [45]. Failure to meet these parameters will render even the best plan unusable. Thus, the venous access catheter–related infection (VACRI) study will deploy a shadow team and a structured approach to gather data that satisfy the rigorous requirements of the CRBSI definition while observing the unconstrained decision-making process, including comparative time and motion analysis [46], of the primary clinical providers. This study will include a convenience sample of all CVAD placements by surgical, medical, nursing, and interventional radiology services at each participating facility, including all high-risk patients referred for central venous access for chronic conditions. In this way, parallel comparisons will be possible between structured clinical management pathways and daily de facto strategies for the most high-need, high medical cost patients. It will also be possible to measure the potential gains or losses in clinical burden that would result from the implementation of the alternate strategies.

Based on the results of COACT I and VACRI, a structured workflow will be designed and implemented in a decision support system that will permit prospective data extraction across the device life cycle, from the time the patient is referred for central catheter insertion until the time of device failure or removal at end of therapy. This clinical decision support will be implemented as part of COACT II. In this study, the intended tip position will be prospectively queried prior to insertion to track the ability of the operator to place the tip in a preferred location, and the patient-oriented outcomes of selected positions will be measured using the validated methodology outlined above. In the preintervention phase, catheter tip position and outcomes will be observed for all qualifying patients. Operators will then be instructed on how to select a target zone (based on prior outcomes analysis) and how to identify the actual location of the catheter tip at the time of insertion. In the postintervention phase, catheter tip position and outcomes will be observed for patients with indwelling CVADs to determine the accuracy with which operators place catheter tips after training, and to assess any improvement in catheter outcomes. A validation study for such training is in progress. The secondary objectives for COACT II are identical to those of COACT I.

#### Infrastructure Development and Evaluation

The VANGUARD CRN infrastructure will be modeled after the optimized workflow and documentation strategies arising from the COACT and VACRI studies. Resulting data may provide access to device, anatomic, and pathologic characteristics for continuing prospective research and surveillance analysis. Insights gained from the CRN will be used to detect early signals of adverse events, improve decision-support systems, facilitate postmarket surveillance, and refine real-time decision-making processes with respect to patient or device selection, diagnosis, and management.

To augment the granularity of device data over the device and disease life span, an auxiliary unique device identification (AUDI) Repository of clinically relevant device characteristics is planned as a part of VANGUARD CRN collaborations with core stakeholders. AUDI metadata will provide a single, accurate data source across the intravascular access medical device domain for research, clinical selection and evaluation, product development, and patient satisfaction. The primary objective of this project is to harmonize interoperability data standards for device identification and characteristics and to provide access to a repository of nonproprietary interoperable device characteristics from sources across the device ecosystem. This repository will contain metadata elements including the unique device identifier (UDI) components, as well as other device features (eg, device type, material, and length), which are not currently standardized among manufacturers and are not included in the Global Unique Device Identification Database but are nonetheless critically relevant to clinical practice and to the improvement of patient-centered outcomes. Selection of the data elements and terminology to be included in the AUDI repository will involve authoritative multistakeholder representation through a Delphi process. As part of this process, a plan must be established for populating the data element values for each identified device and for curating the repository. The inclusion of these data elements into the AUDI repository will contribute significant value toward the improvement of semantic interoperability for clinically relevant characteristics of CVADs [47].

The current state of semantic interoperability demands improvement, including expansion, alignment, and usage of data standards, for CVAD terminology usage for patients across many different care settings. The VANGUARD CRN Interoperability Standards project comprises several foci, including venous access clinical terminology, venous access device metadata, and related vascular anatomy. There are several factors to address (1) the identification of key components of care and early signals of infectious and other complications and their impact on patient needs and costs over time, (2) the identification of clinically relevant device characteristics that may contribute to the understanding of patient outcomes, especially when infections or other complications are present, and (3) accurate identification of the anatomical placement of the CVAD in the patient and of significant venous events such as vessel clotting or narrowing. To address the first focus, VANGUARD continues to work within the HL7 (Health Level Seven) community and with other stakeholders to harmonize terms, refine logical data models, and translate results into Fast Health Care Interoperability Standards profile definitions and implementation guides.

The AUDI project aims to address the improvement of the second semantic interoperability objective, but there are several concerns regarding device data availability. Foremost, some data are distributed across data sources that are not computationally accessible (eg, manufacturer databases, labels, instructions for use, and public websites) or is inconsistently formatted across manufacturers or device types. An example of this is whether, for a catheter with multiple channels, if the number of lumens is recorded at all, it is recorded as a numeric value (eg, 2 and 3) or as a text-based value (eg, dual vs double or triple lumen). Inconsistencies in recording such factors across data sources may lead to the inaccurate linkage of key auxiliary device data. A consolidated and verified data source would mitigate many potential inconsistencies [48,49]. There are ongoing efforts to use artificial intelligence, machine learning, and matching algorithms to expedite terminology mapping and standardization, although issues of cost and access remain.

To address the third focus, the VANGUARD CRN Vascular Anatomy project will develop a structured interactive graphical interface for an interoperable anatomy atlas for standards-based terminology, location, function, and characterization of intravascular events. This will provide a point-and-click interface for the clinician to document the insertion of a catheter by scanning the device label and scanning the patient ID, and for other clinicians, stakeholders, and the patients themselves to access the patient’s venous health history in a single searchable reference image. Linking this resource to the device and to the patient through the use of a unique device identifier (will provide critical continuing care documentation across time and venue of the patient’s venous health and intervention history. Clinical outcomes data such as the occurrence of venous obstruction, catheter-related infections, mechanical complications, and catheter-related mortality gathered from the COACT I and II studies will be integrated with device and location data in the vascular anatomy atlas to improve modeling of decisions and patient-centered outcomes.

To meet its core objectives, the VANGUARD initiative cannot rely solely on clinician-driven research. It is vital to understand and address meaningful components of care and accurately measured outcomes derived from and validated by patients, families, and patient representatives [10]. Because a considerable proportion of central venous access care and complications occur outside hospitals and beyond the reach of EHRs and national surveillance systems, the only stakeholder routinely aware of critical CVAD-related events across time and venues may be the patient. Even within the hospital, the patient has a singular interest in the accuracy of data gathering and the effectiveness of diagnosis and management. Yet, the patient often feels excluded from medical decision-making. Efforts toward self-advocacy are often dismissed by busy and distracted care providers; increasing patient assertiveness or willingness to challenge providers or other medical authority and to actively participate in decision-making to ensure they receive the treatment they feel best meets their needs can sustainably improve health care outcomes and reduce costs [50,51]. For these reasons, the VANGUARD CRN is working toward the collaborative development of patient-facing tools to allow patients to exercise their rights to use and share their own health data, to facilitate their ability to provide self-generated input, as well as to support and leverage their participation in health care decision-making [52-54]. A VANGUARD CRN Patient-Facing Platform will help to improve data completeness and validity and to empower patient self-advocacy.

## Discussion

Currently, when it comes to assessing the real-world performance of CV access devices, there is a plenitude of low-quality data that contribute little to understanding the relationship between elective clinical decisions and critical outcomes relevant to patients.. The initial VANGUARD studies will be focused on data collected from CVAD patients in the controlled environment of medical institutions, where there should be routine documentation of vital signs taken, laboratory tests obtained, medications administered, and CVADs placed or removed. Given the context of inpatient care and access to clinical resources, additional prospective studies may be needed to fully understand the issues encountered in outpatient or emergent care settings. The roadmap presented here reflects the anticipated need for an iterative approach to these studies.

One of the challenges arising from the current state of semantic interoperability is the lack of domain-specific vocabulary standards for venous access patients. To facilitate the Interoperability Standards project, one universal standard language of medical terminology must be adopted across stakeholders including all EHR and other health information technology platforms. The definitions, settings, and treatment of infectious diseases (such as CLABSI and CRBSI) cannot be debatable due to variations in language interpretation across stakeholder silos. One standard lexicon must be achieved, whether it be newly curated medical terminology or an improvement of the existing national standards. Semantic interoperability will be paramount, and its achievement will frame the infrastructure of the future [55,56].

Lastly, the problem of addressing discrepancies between documented and factual infection rates is complicated by the limitations of existing data. For example, studies of health care–associated infections report that critical laboratory results or medication administration may not exist in structured databases if a patient has received care outside the index hospital. This type of missing information varies widely across different health organizations and is especially prevalent in tertiary medical centers where patients commonly travel a long distance to receive care [57]. Moreover, the infection may not be recorded due to the lack of laboratory testing either within or outside of the index hospital, and therefore these records will be overlooked in surveillance for catheter-related complications.

Similar circumstances are common across the health care ecosystem: a wealth of data with critical gaps and impairments of meaning. There are fundamental shortcomings in the way data is documented, extracted, and exchanged that magnify the disparity between data volume and meaning, including lack of interoperability and friction in the integration of data sources [55-57]. These shortcomings do not serve the needs of patients or the national interest in the provision of safe and effective care. In addition, the entire clinical interoperability ecosystem must be set on a strong foundation of standards-based observations to accurately capture patients’ experiences and outcomes. A systematic approach to the representation of clinical knowledge and the careful integration of domain-specific language is essential for the meaningful application of electronic knowledge management systems to clinical decision-making and care improvement [58]. VANGUARD aims to demonstrate the value and translational potential of this composite knowledge interoperability approach. The VANGUARD CRN initiative is targeting a recognized national priority, catheter-related complications, in high-need, high-cost populations. The intent of this initiative is to create a roadmap to measurable improvements in the delivery of care, sensitive to the prevailing clinical workflow and documentation burden that will address ecosystem-wide issues and will be useful to others in the CRN Learning Community and meaningful to the advancement of patient health and well-being.

## Abbreviations

AUDI: auxiliary unique device identification
CLABSI: central line–associated bloodstream infection
COACT I: Clinical Outcome Analysis of Catheter Tip-Position
CRBSI: catheter-related bloodstream infection
CRN: Coordinated Registry Network
CVAD: central venous access device
EHR: electronic health record
HL7: Health Level Seven
MDEpiNet: Medical Device Epidemiology Network
RCP: Research Consensus Panel
VACRI: venous access catheter–related infection
VANGUARD: Venous Aaccess: National Guideline and Registry Development

## Data Availability

Data sharing is not applicable to this paper as no data sets were generated or analyzed during this study.
